# The effectiveness of Stepping Stones Triple P parenting support in parents of children with borderline to mild intellectual disability and psychosocial problems: a randomized controlled trial

**DOI:** 10.1186/s12916-014-0191-5

**Published:** 2014-10-28

**Authors:** Marijke Kleefman, Daniëlle EMC Jansen, Roy E Stewart, Sijmen A Reijneveld

**Affiliations:** Department of Health Sciences, University Medical Center Groningen, University of Groningen, PO Box 196, 9700 AD Groningen, the Netherlands; Department of Sociology and Interuniversity Center for Social Science Theory and Methodology (ICS), University of Groningen, Grote Rozenstraat 31, 9712 TG Groningen, the Netherlands

**Keywords:** Borderline to mild intellectual disability, Children, Psychosocial problems, Parenting support, Randomized controlled trial

## Abstract

**Background:**

Children with borderline to mild intellectual disability (BMID) have been shown to be at increased risk for psychosocial problems. The presence of these psychosocial problems leads to parenting stress. Stepping Stones Triple P (SSTP) is a parenting support program to support parents with children with BMID and psychosocial problems. The aim of this study was to evaluate the effectiveness of SSTP compared to Care as Usual (CAU) in reducing psychosocial problems in children with BMID.

**Method:**

We conducted a randomized controlled trial in the Northern provinces of the Netherlands. Parents of children aged 5 to 12 with borderline (IQ 70 to 85) or mild (IQ 70 to 50) ID and psychosocial problems were invited. Psychosocial problems were identified using the Strengths and Difficulties Questionnaire (SDQ) parent report (≥14). Measurements were assessed before the intervention (T0), immediately after the intervention (T1) and after a follow-up of six months (T2). SSTP takes 8 to 10 individual sessions of 40-90 minutes, provided over 10 to 12 weeks. CAU concerned any service, except SSTP. Primary outcomes were the child’s psychosocial problems (SDQ parent and teacher forms and the Eyberg Child Behavior Inventory, ECBI). Secondary outcomes were parenting stress (Parenting Stress Index, PSI) and parenting skills (Alabama Parenting Questionnaire, APQ).

**Results:**

In total 209 parents of children aged 5 to 12 with BMID were allocated blindly to either SSTP (n =111) or CAU (n =98). In the intention to treat analyses, SSTP achieved no significantly better effect than CAU for the SDQ parent report, the ECBI and the APQ on the short- and long- term. In the short term, SSTP was significantly more effective than CAU for the SDQ teacher report (B = -2.25, 95% CI -3.79 to -0.71) and the PSI (B = -7.06, 95% CI -12.11 to -2.01). For both SDQ teacher report and PSI, there was no statistically significant effect in the long term. Dropout from SSTP was considerable (49%), with the effects being solely found in the adherent SSTP subgroup.

**Conclusions:**

SSTP had some short-term advantages over CAU, but not in the longer term.

**Trial registration:**

Dutch Trial Register NTR2624. Registered 26 November 2010

## Background

Psychosocial problems, such as problems with behavior and emotions, occur frequently in children with borderline to mild intellectual disability (BMID) [1]. Prevalence rates vary widely, from 30% to more than 60% [1-3]. The combination of psychosocial problems and BMID is likely to restrict school and social participation and can also limit occupational opportunities in the post-school period [4]. Furthermore, raising a child with BMID and psychosocial problems is likely to lead to parenting stress [5-7]. The child’s psychosocial problems and parenting stress are likely to exacerbate each other over time [8].

Improving parenting skills using parenting interventions has been shown to lead to great reductions in both the child’s psychosocial problems and the parents’ parenting stress [9]. A promising parenting programme is Stepping Stones Triple P (SSTP). SSTP is part of the Australian Triple P, Positive Parenting Programme. This program is a family intervention that aims to prevent and reduce severe behavioral, emotional and developmental problems in children with all kinds of disabilities, including BMID, by enhancing the knowledge, skills and confidence of parents [10,11].

Although SSTP seems promising, evidence of its effectiveness is very scarce. Results of some studies in Australia showed significant improvements in child behavior and parenting styles in different target populations of pre-school children, children with autism or other developmental disabilities [12-15]. In addition, a Dutch non-randomized, non-controlled study of SSTP has shown positive effects on psychosocial problems in children, on parenting skills, family functioning and parental wellbeing [16]. However, these findings have been challenged on the basis of a number of weaknesses. First, the Australian developers were involved in all the effectiveness studies. Second, these studies had small sample sizes or comprised children without BMID. Furthermore, many of these studies did not compare the effects with other interventions offered simultaneously or Care as Usual (CAU) [17].

Accordingly, convincing evidence of the effects of individual SSTP in children with BMID and their parents is still lacking. Therefore, the aim of this study was to assess the effectiveness of the SSTP parenting support program in reducing psychosocial problems in children with BMID compared to CAU.

## Methods

### Research design

The study was conducted as a randomized controlled trial with three assessments: before the intervention (T0), immediately after the intervention (T1) and six months later (T2), and is reported following the CONSORT guidelines [18]. Full details of the trial protocol can be found elsewhere [19]. The Medical Ethics Committee of the University Medical Center Groningen approved the study design. Parents participated voluntarily in this study, having signed to attest their informed consent and were free to leave the study at any time.

### Study setting and participants

We obtained a sample of parents using a two-step process. First, through schools, parents of children 5- to 12-years old with borderline (IQ 70 to 85) or mild (IQ 70 to 50) intellectual disability (ID), living in the four northern provinces of the Netherlands (Groningen, Friesland, Drenthe and a part of Overijssel) were invited to complete a screening measurement (that is, T0) about their child’s psychosocial problems and their parenting skills. In the Netherlands, children 5- to 12-years old with BMID mainly attend three types of schools for special educational needs, known in Dutch as SBO, REC3 and REC4. SBO (Speciaal Basis Onderwijs: special primary education) includes children with borderline intellectual disabilities (IQ 70 to 85), learning difficulties and/or behavior difficulties. REC 3 (Regional Expertise Center cluster 3) is a type of school for children with physical disabilities, mild to severe intellectual disabilities (IQ <55 or IQ 56 to 70 with other severe disabilities) and/or chronic diseases. REC 4 (REC cluster 4) serves children with psychiatric and/or behavioral disorders with borderline ID (IQ 70 to 85) or children with psychiatric and/or behavioral disorders without ID (IQ >85) [20,21]. All the participating parents completed the Strengths and Difficulties Questionnaire (SDQ) about their child’s psychosocial problems [22,23]. To increase response rates, schools sent a reminder to all parents who did not respond within four weeks and each school published a newsletter on the study, based on information provided by the researchers.

In the second step of sampling, eligible parents of children with a clinical Total Difficulties Score (TDS) on the SDQ parent form of 14 or higher were invited by the researcher to participate in the intervention study. If necessary, we offered assistance in completing the screening measurement (T0). Moreover, parents completed the second and third questionnaires during a visit by a research assistant, who was thereby able to provide assistance.

### Exclusion

At the first step of the sampling process, the screening exclusion criteria were: (1) the child lived in residential care (except foster care); (2) the parents were unable to speak Dutch; (3) information about the child’s IQ was not available; or (4) the parents lived outside the research area. At the second step, the intervention selection, the exclusion criteria were: (1) a brother or sister (with a higher SDQ-TDS) was already participating in the study: and (2) the parents were receiving treatment for parenting skills or other treatment that potentially conflicted with SSTP.

### Intervention

SSTP aims to enhance the knowledge, skills and confidence of parents to prevent behavioral, emotional and developmental problems in children with disabilities, including BMID [11]. SSTP is based on seven key steps to positive parenting: (1) ensure a safe, interesting environment; (2) create a positive learning environment; (3) use assertive discipline; (4) have realistic expectations; (5) take care of oneself as a parent; (6) family adaptation to having a child with a disability; and (7) be part of the community. The last two principles are specific extensions of Triple P for Stepping Stones, targeting the specific problems of raising children with a disability [13].

SSTP requires eight to ten individual sessions of 40 to 90 minutes each, divided over four modules and provided over a period of ten to twelve weeks. The first module, ‘Assessment’, consists of two sessions of about 60 to 90 minutes each. In this module, the parents formulate hypotheses about the problems and make relevant causes and factors clear. The second module, ‘Positive Parenting’, also consists of two sessions of about 60 to 90 minutes. These sessions introduce parenting strategies to the parents. The third module, ‘Practice’, consists of three sessions of about 40 to 60 minutes each. In these sessions, parents practice their newly acquired parenting strategies and receive support. The final module, ‘Planned Activities Training’, consists of three sessions of about 60 to 90 minutes. In these sessions, parents are assisted in the practical implementation of the strategies [11].

In this study, eight SSTP health care professionals delivered SSTP. These professionals were all SSTP accredited, that is, they completed training by an accredited SSTP trainer and an accredited SSTP trainer provided periodic supervision. The professionals worked for a Dutch healthcare organization that specialized in clients with disabilities (Dutch: MEE). These professionals did not provide CAU.

### Care as Usual

Parents assigned to the control condition, CAU, could use any service except SSTP. The main types of service used were Practical Pedagogical Family Support (PPG), Video-home training (VHT), Intensive Pedagogical Homecare (IPT) or Intensive Orthopedagogical Family Care (IOG), but individual psychiatric or psychological care for the child was also sought and in some cases no care at all.

### Primary outcomes

The primary outcomes of the study were child’s psychosocial problems, measured with the SDQ on the parent and teacher forms and with the Eyberg Child Behavior Inventory (ECBI). The SDQ consists of questions on four subscales with five items each: emotional symptoms, conduct problems, hyperactivity and peer relationship problems. Each item can be scored on a 3-point scale (0 = not true, 1 = somewhat true and 2 = certainly true), yielding a TDS ranging from 0 to 40 [22,23]. The ECBI consists of 36 items in which parents rate how often behavior occurs. Each item can be scored on a 7-point scale (1 = ‘never’ to 7 = ‘always’). The sum of these scores yields a sum score on the ECBI ranging from 36 to 252 [24,25].

### Secondary outcomes

The secondary outcomes were parenting practices and stress. Parenting practices were measured using the Alabama Parenting Questionnaire (APQ), short version [26]. This consists of 35 items on parenting practices, in four subscales: parental involvement, positive parenting, poor monitoring and inconsistent discipline. Each item can be scored on a 5-point scale (1 = ‘never’ to 5 = ‘always’) which yields a sum score on the APQ ranging from 35 to 175 [26,27].

Parenting stress was measured using the short Dutch version of the Parenting Stress Index (PSI) [28]. This questionnaire consists of 25 statements about experiences related to child characteristics, parent characteristics and situations that are directly related to the role of being a parent. Each item can be scored on a 6-point scale (1 = totally disagree to 6 = totally agree) which yield together a sum score on the PSI ranging from 25 to 150 [28].

### Background characteristics

Background characteristics concerned the gender and age of the child, ethnicity (both Dutch or one or both non-Dutch), parental education, parental employment and family composition. Parental education was categorized as: 1) low education: elementary or lower levels of secondary education; 2) middle: higher levels of secondary education or intermediate vocational education; 3) high: higher vocational education and university education. Parental employment was categorized as 1) yes: if at least one parent worked more than 12 hours a week and 2) no: if both parents together worked fewer than 12 hours a week. Family composition was categorized as 1) two biological parents and 2) other: one parent, co-parents, adoption and foster parents.

The treatment integrity was measured by the number of sessions attended by the parents. SSTP was completed adequately if the family had attended at least five sessions.

### Sample size

The parental SDQ-TDS served as the primary outcome measure for determining the sample size needed. For a three-point decrease in the SDQ-TDS, given a standard deviation (SD) for the SDQ of six points (that is, an effect size of 0.5), at alpha =0.05 (two-sided) and beta =0.20, 63 children needed to be included in each group (SSTP and CAU). With adjustment for an estimated ‘loss to follow-up’ of 40%, 210 children needed to be included in the study, 105 children in each treatment condition.

The prevalence rate of SDQ-TDS ≥14 in BMID children not under current treatment for their mental health problems was estimated at 55% [1]. Therefore, (2 × 105)/55% =381 children with BMID, 5- to 12-years old, were required. Accounting for 30% refusal to participate at that step and 10% incomplete SDQs, 635 parents were need to complete the SDQ at the first step of the selection procedure.

### Randomization

Eligible individuals were randomized per center in each of the four participating centers (Groningen, Friesland, Drenthe and Overijssel) in mixed blocks of four and six to prevent unequal randomization within the centers [29], using a computer-generated randomization algorithm. If parents were randomized to SSTP, the intervention started within four weeks of administering the screening measurement (T0). In the first part of the study the randomization ratio was 1 to 1. After one year of inclusion, this was adapted to 2 SSTP to 1 CAU because of a relatively high drop-out rate in the SSTP group.

### Blinding

Parents were allocated blindly to either SSTP or CAU. Furthermore, the teachers did not know who was participating in which group. The SSTP trainers could not be blinded to the treatment status during the intervention. Last, the research assistant was also not blinded to treatment status.

### Statistical analyses

First, we described the flow of participants – parents – in a diagram [30].

Second, we described the baseline characteristics of the parents in each research group. The differences between the groups were tested using Pearson Chi-square tests for categorical variables and one-way analysis of variance (ANOVA) for continuous variables.

Third, we compared the effectiveness of SSTP with CAU on the primary and secondary outcomes by assessing the effects of SSTP compared to CAU using mixed model techniques. In this analysis, the individual measurements were the first level and the child was the second. The effects on the SSTP group compared to the CAU group were assessed as regression coefficients (B) with 95% confidence intervals (CI) in the short term (that is, T0 to T1) and in the long term (that is, T0 to T2) adjusted for age and gender. First, we performed an intention to treat (ITT) analysis, in which all randomized parents were analyzed regardless of whether or not they completed the intervention and any post-treatment questionnaire, with the last observation carried forward. Because of the high dropout rate in the SSTP group, this group was also split into SSTP completed and SSTP not completed for additional analyses.

Finally, all the parents who completed the screening measurement and at least one post-measurement were included in the complete case analyses. All analyses were performed using SPSS Statistics version 20.0.

### Ethical permission

Ethical permission for this study was obtained from the Medical Ethics Committee of the University Medical Center Groningen (METc2010.203; ABR: NL29554.042.10). All participants gave informed consent before taking part in the study.

## Results

**Figure 1:**
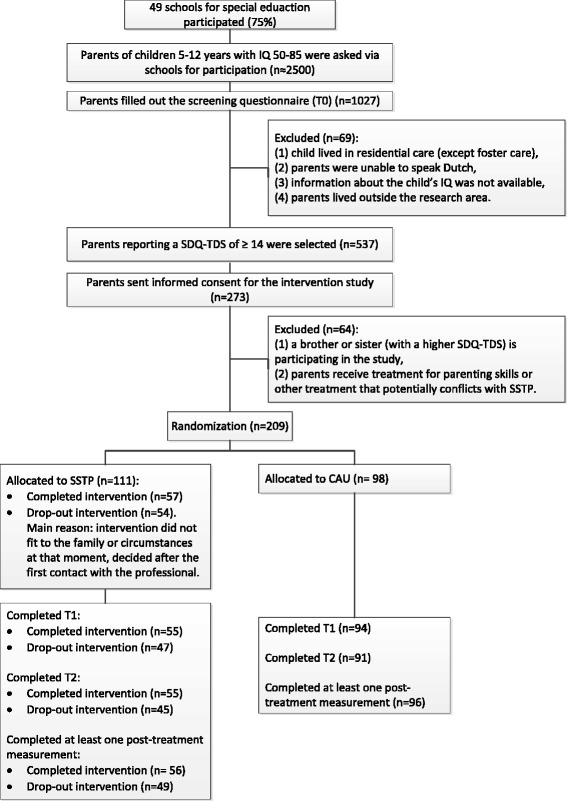
**Flow of participants through the study.**

The study was performed between October 2010 and October 2013. Figure 1 shows the flow of participants through the study. A total of 49 schools (75% of those invited) agreed to participate. The main reasons for school non-participation were participating in other research, being under increased monitoring by the superintendent of schools and having too little time. Non-participating schools did not differ from participating schools in terms of location (rural or urban) and type. A total of 1,027 parents completed the screening measurement (T0).

After selection and randomization, 209 parents were randomized to either CAU (n =98) or SSTP (n =111) at the second step of the sampling procedure. All initial 209 parents were included in the ITT analysis. For complete case analyses, data on at least one post-treatment measurement was available for 201 parents (n =105 in SSTP and n =96 in CAU). Of the SSTP group, data were available on 56 parents who adequately completed SSTP (that is, attended at least five sessions) and on 49 parents who did not adequately complete SSTP (that is, attended fewer than five sessions).

### Baseline data

At baseline, SSTP and CAU groups did not differ regarding any background variable other than parental employment; fewer parents were unemployed in the SSTP group compared to the CAU group (*P* <0.05). There were no significant differences between the two groups for any of the outcome variables at baseline. This indicates that the randomization procedure generally resulted in two similar groups. Furthermore, SSTP completed, SSTP not completed and CAU groups did not differ from each other either in terms of any of the background or outcome variables. Table 1 shows the baseline characteristics of the parents in all the groups.

**Table 1:** **Baseline characteristics (mean (SD) or %) of participants by treatment group (n =209)**

**Characteristics**	**SSTP**			**CAU**	***P-*** **value** ^**a**^	***P-*** **value** ^**b**^
	**Completed**	**Not completed**	**Total SSTP**			
Number	57	54	111	98		
Child						
Age of the child	9.70 (2.07)	10.13 (1.60)	9.91 (1.86)	9.65 (2.01)	0.337	0.319
Gender of child (boys)	64.9%	50.0%	57.7%	58.2%	0.941	0.282
Ethnicity Dutch	94.7%	94.4%	94.6%	99.0%	0.082	0.212
Parents						
Mother’s education (medium-high)	61.4%	48.2%	54.9%	54.0%	0.322	0.386
Father’s education (medium-high)	56.6%	42.0%	49.5%	44.7%	0.408	0.488
Employed (>12 hours/week)	92.0%	93.8%	92.2%	83.3%	**0.042**	0.123
Family						
Two-parent family	68.4%	69.8%	69.1%	64.3%	0.463	0.754
Pre-measures						
SDQ_p (score 0-40 /tds)	19.00 (4.33)	19.52 (4.33)	19.25 (4.32)	19.84 (4.00)	0.313	0.486
SDQ_t (score 0-40 /tds)	14.96 (7.05)	13.98 (6.18)	14.46 (6.61)	13.50 (6.69)	0.340	0.498
ECBI (score 36-252 /ss)	131.30 (27.64)	119.80 (25.61)	125.70 (27.17)	127.67 (27.19)	0.605	0.072
PSI-s (score 25-150 /ss)	79.40 (24.79)	68.64 (24.64)	74.22 (25.19)	72.82 (23.95)	0.682	0.065
APQ (score 35-175 /ss)	99.72 (7.89)	98.89 (8.10)	99.32 (7.97)	98.80 (8.39)	0.651	0.783

^a^Differences tested between SSTP and CAU groups at baseline; ^b^differences tested between SSTP completed, SSTP not completed and CAU groups at baseline. APQ, Alabama Parenting Questionnaire; CAU, Care as Usual; ECBI, Eyberg Child Behavior Inventory; PSI-s, Parenting Stress Index – short version; SD, standard deviation; SDQ_p, Strengths and Difficulties Questionnaire parent version; SDQ_t, Strengths and Difficulties Questionnaire teacher version; ss, sum score; SSTP, Stepping Stones Triple P; tds, total difficulties score.

### Treatment integrity

The number of sessions parents who were randomized to SSTP received varied from zero to ten. In the SSTP completed group, the number of sessions varied from five to ten. None of the parents in the SSTP group received additional support during the period of receiving SSTP. Fifty-four of the 111 parents were in the SSTP not completed group (49%). Of these 54 parents, 34 did not start the intervention after the intake and 20 parents did not finish before completing at least five sessions. Their reasons for dropout included family circumstances (divorce, financial problems or sickness), starting another comparable parenting support intervention, parental expectations that the intervention would be too intensive, lack of time, or the parents’ non-recognition of their child’s psychosocial problems. Of these 54 parents, 25 (46%) started another parenting intervention. In the CAU group, 36 parents indicated that they received parenting support such as phone contact with a professional, a home visit by a professional, intervention ‘Intensive Pedagogical Homecare (IPT)’, or intervention ‘Intensive Orthopedagogical Family Care (IOG)’. In this CAU group, 62 parents did not receive parenting support during the study period.

### Effects on primary and secondary outcomes

Table 2 presents the effects based on the ITT analyses of all 209 parents regarding SSTP compared to CAU in the short term (T0 to T1) and in the long term (T0 to T2). All analyses were adjusted for gender and age. Regarding the SDQ parent form, SSTP did not differ significantly from CAU in the short term. Parents in the SSTP group scored lower on the SDQ in the short term than parents in the CAU group (B = -0.05, 95% CI -1.23 to 1.12); this difference was not statistically significant. The differences between the two groups remained non-significant in the long term (B =0.06, 95% CI -1.12 to 1.24). Furthermore, no differences were found on the ECBI between the two groups in the short and long term. On the SDQ teacher form, teachers in the SSTP-group did differ significantly from those in the CAU group after completion of the intervention (short-term). Teachers in the SSTP group scored children lower on the SDQ than teachers in the CAU group in the short term (B = -2.25, 95% CI -3.79 to -0.71). However, no significant differences between SSTP and CAU were found on the SDQ teacher form in the long term.

**Table 2:** **Raw means at T0, T1 and T2 and regression coefficients based on intention to treat analyses with mixed models (n = 209)**

**Outcome**	**Group**	**T0 (Baseline)**	**T1 (three months or immediately after intervention)**	**Difference in improvement T0 to T1 between SSTP and CAU**		**T2 (six months after intervention)**	**Difference in improvement T0 to T2 between SSTP and CAU**	
		**Mean (SD)** ^**a**^	**Mean (SD)** ^**a**^	**B (95% CI)** ^**b**^	***P*** **-value** ^**b**^	**Mean (SD)** ^**a**^	**B (95% CI)** ^**b**^	***P*** **-value** ^**b**^
SDQ_p^c^	SSTP	19.23 (4.38)	17.28 (5.64)	-0.05 (-1.23; 1.12)	0.927	17.31 (5.62)	0.06 (-1.12; 1.24)	0.916
	SSTP-c	18.98 (4.36)	16.98 (5.88)	-0.29 (-1.70; 1.12)	0.689	16.60 (5.57)	-0.27 (-1.68; 1.14)	0.706
	SSTP-nc	19.51 (4.43)	17.64 (5.40)	0.19 (-1.25; 1.63)	0.794	18.18 (5.62)	0.44 (-1.02; 1.89)	0.557
	CAU	19.85 (4.00)	17.93 (5.34)			18.01 (5.12)		
SDQ_t^c^	SSTP	14.52 (6.70)	13.96 (6.50)	-2.25 (-3.79; -0.71)	**0.004**	13.09 (5.12)	-1.10 (-2.65; 0.46)	0.165
	SSTP-c	14.96 (7.05)	14.46 (6.98)	-2.16 (-4.04; -0.28)	**0.025**	13.61 (4.67)	-1.39 (-3.28; 0.50)	0.149
	SSTP-nc	14.05 (6.34)	13.43 (6.01)	-2.34 (-4.22; -0.47)	**0.014**	12.46 (5.63)	-0.80 (-2.71; 1.11)	0.410
	CAU	13.59 (6.66)	14.60 (6.46)			13.33 (7.19)		
ECBI^c^	SSTP	126.36 (27.60)	113.85 (28.03)	-4.71 (-10.05; 0.63)	0.084	114.37 (27.94)	-3.83 (-9.20; 1.55)	0.163
	SSTP-c	131.90 (27.49)	116.62 (27.76)	-8.83 (-15.22; -2.44)	**0.007**	116.84 (26.84)	-6.15 (-12.55; 0.24)	0.059
	SSTP-nc	120.02 (26.59)	110.54 (30.48)	-0.37 (-6.86; 6.13)	0.912	111.36 (29.25)	-1.38 (-7.98; 5.21)	0.680
	CAU	127.63 (27.35)	120.51 (24.55)			118.07 (2.77)		
PSI-s^d^	SSTP	74.68 (25.71)	66.67 (24.65)	-7.06 (-12.11; -2.01)	**0.006**	66.57 (26.37)	-3.19 (-8.28; 1.89)	0.217
	SSTP-c	79.34 (25.01)	68.35 (22.35)	-10.42 (-16.46; -4.37)	**0.001**	69.47 (26.65)	-4.62 (-10.67; 1.43)	0.134
	SSTP-nc	69.25 (25.70)	64.62 (27.31)	-3.45 (-9.63; 2.74)	0.274	63.02 (25.88)	-1.69 (-7.96; 4.58)	0.596
	CAU	73.14 (24.02)	72.46 (22.93)			68.14 (23.31)		
APQ^d^	SSTP	99.54 (7.95)	99.10 (9.17)	-1.27 (-3.52; 0.98)	0.267	101.21 (10.87)	1.23 (-1.03; 3.49)	0.286
	SSTP-c	99.89 (7.05)	98.65 (7.52)	-1.68 (-4.37; 1.02)	0.223	101.59 (11.55)	1.30 (-1.40; 4.00)	0.346
	SSTP-nc	99.14 (8.12)	99.63 (10.87)	-0.84 (-3.59; 1.90)	0.546	100.78 (10.17)	1.13 (-1.65; 3.91)	0.423
	CAU	98.58 (8.33)	99.20 (9.21)			98.26 (9.75)		

^a^Raw mean scores on the different outcome measurements; ^b^B for SSTP compared to CAU, based on mixed model techniques**,** expressing differences in change between SSTP and CAU in outcomes – analyses were adjusted for gender and age; ^c^primary outcome; ^d^secondary outcome. APQ, Alabama Parenting Questionnaire; B, Regression Coefficients; CAU, Care as Usual; CI, confidence interval; ECBI, Eyberg Child Behavior Inventory, PSI-s, Parenting Stress Index short version; SD, standard deviation; SDQ_p, Strengths and Difficulties Questionnaire parent version; SDQ_t, Strengths and Difficulties Questionnaire teacher version; SSTP, Stepping Stones Triple P; SSTP-c, Stepping Stones Triple P completed; SSTP-nc, Stepping Stones Triple P not completed.

Regarding the secondary outcome parenting stress (PSI), the SSTP and CAU groups differed significantly after completion of the intervention (that is, short-term). Parents in the SSTP group scored lower on the PSI than parents in the CAU-group in the short term (B = -7.06, 95% CI -12.11 to -2.01). However, no significant differences were found on the PSI in the long term. Finally, no differences were found between the SSTP and CAU groups in either the short and long term on the APQ.

Second, effects of time were analysed. In both the CAU-group and the SSTP-group, the PSI decreased significantly over time (*P* =0.009). On the primary outcomes, SDQ parent and teacher version and ECBI, and on the secondary outcome APQ we found no statistically significant differences over time.

Third, we repeated all analyses in three groups to compare the effects of both SSTP completed and SSTP not completed with CAU in the short and long term. With respect to the SDQ parent and teacher form, neither SSTP completed nor SSTP not completed resulted in statistically significant different outcomes compared to CAU after completion of the intervention (T0 to T1) and at the six-month follow-up (T0 to T2). Significant differences were found between the SSTP completed and CAU groups in the short term on the SDQ teacher form, ECBI and the PSI. No differences were found for the other measurements (see Table 2).

Finally, we performed complete case analyses on 201 parents who completed at least one post measurement. These did not reveal any statistically significant differences between the SSTP and CAU groups for any of the outcome measures (results not shown).

## Discussion

This study evaluated the effectiveness of the parenting support program SSTP compared to CAU in reducing psychosocial problems in children with BMID. The parents of children with clinical psychosocial problems (SDQ-TDS ≥14) were included. In both the ITT and complete case analyses, we found significant differences between SSTP and CAU in the short term for the SDQ teacher form and PSI, but not in the long term. We found no significant differences in effects between SSTP and CAU on the other primary or secondary outcomes at either post-intervention measurement.

We found some advantages in the short term for SSTP over CAU, but no advantages in the longer term. These findings contrast with previous studies which found more positive effects for SSTP on a child’s psychosocial problems, on parenting skills, family functioning and parental wellbeing [12-16]. Several explanations for this difference in findings can be provided. First, we compared the SSTP with a control group CAU, whereas other studies only compared SSTP with a waiting list group or no control at all [12-14,16]. Second, our study included parents from schools for special education who were selected for intervention using a screening measurement for psychosocial problems. Previous studies included parents who were explicitly referred to healthcare because of problems experienced in daily life or which focussed on children with specific problems, such as autism or physical disabilities [12,14-16]. It is reasonable to expect that the effects of the intervention would be different, because parents in previous studies had already perceived a need for treatment.

Third, other studies which reported the effectiveness of SSTP were either performed by its developers in Australia or had small sample sizes [12-15]. Therefore, those studies should be interpreted carefully due to information and selection bias [31]. Finally, in contrast with other studies, we used an independent data collection process, which meant that parents were asked to complete questionnaires in the absence of the health care professional who was carrying out the intervention. These questionnaires were not specifically developed for or used in the SSTP intervention.

In summary, our study was carried out effectively and designed to a high standard, owing to a sufficient sample size, independent data collection and control group, which strengthens our findings.

### Strengths and limitations

The main strengths of our study have already been indicated. First, randomization prevented selection and allocation bias, meaning that its internal validity is high. Second, we recruited from schools for special education, so the majority of parents of children with BMID were reached [20]. This increases the external validity and generalizability of our results [32]. Third, our study was well powered because the sample size was sufficient. Fourth, our study had a low loss to follow up because parents received assistance by an independent research assistant in completing the questionnaire if they participated in the intervention study. Furthermore, our data collection was fully independent of the intervention itself, to ensure that the overview obtained was more objective and to avoid social desirability bias [17]. Sixth, we had a follow-up measurement six months after the intervention to enable the study of the effects of the SSTP on psychosocial problems over time. A final strength was the use of two informants to measure the child’s psychosocial problems (that is, teacher and parent). More informants lead to a better understanding of a child’s functioning, because psychosocial problems can be highly situational and differ at school and at home [33-35].

Our study also had an important limitation: there was selective dropout in the intervention group. Of the 111 randomized parents in the SSTP group, only 57 completed the intervention. Parents in the group that completed SSTP reported more baseline problems on the ECBI (child’s behavior problems) and the PSI (parenting stress) than parents in the group that did not complete SSTP.

## Conclusions

This study found some effects in the short term but no effects in the long term for SSTP compared to CAU over time and across outcomes. In terms of the child’s psychosocial problems at school and the parents’ parenting stress, we found significant differences between the SSTP and CAU groups immediately after the intervention. However, we found no differences six-months after treatment for those two outcomes, nor for the other outcomes.

This study had a high drop-out rate in the SSTP group. The reasons for this deserve additional study as SSTP may not fit this population despite being promising in theory. SSTP might be too intense for certain populations, or insufficiently intense because of the number of problems parents have to deal with [36-38]. Another implication for research is to evaluate the costs of SSTP compared to CAU to determine whether implementation of the SSTP yields cost benefits [39,40].

## What is already known on this topic

Stepping Stones Triple P seems to be a promising intervention for the parents of children with borderline to mild intellectual disability. However, evidence of its effectiveness remained weak.

## What this study adds

This study adds evidence of the effects of SSTP compared with CAU in a randomized controlled trial. Its results show some short-term advantages and no long-term advantages for SSTP compared to CAU over time and across outcomes.

## Abbreviations

APQ: Alabama Parenting Questionnaire
BMID: Borderline to mild intellectual disability
CAU: Care as Usual
CI: confidence intervals
ECBI: Eyberg Child Behavior Inventory
ITT: intention to treat
PSI: Parenting Stress Index
REC: Regional Expertise Center
SBO: Speciaal Basis Onderwijs, special primary education
SD: standard deviation
SDQ: Strengths and Difficulties Questionnaire
SPSS: Statistical Package for the Social Sciences
SSTP: Stepping Stones Triple P
TDS: Total Difficulties Score
ZonMW: Netherlands Organization for Health Research and Development
